# Necroptosis in bacterial infections

**DOI:** 10.3389/fimmu.2024.1394857

**Published:** 2024-06-12

**Authors:** Xing Yu, Jin Yuan, Linxi Shi, Shuying Dai, Lei Yue, Min Yan

**Affiliations:** ^1^ Department of Pathogen Biology and Immunology, Faculty of Basic Medical Science, Kunming Medical University, Kunming, China; ^2^ Clinical Laboratory, Puer Hospital of Traditional Chinese Medicine, Puer, China; ^3^ Institute of Medical Biology, Chinese Academy of Medical Sciences and Peking Union Medical College, Kunming, China

**Keywords:** necroptosis, bacterial infection, inflammatory cells, ripk1, ripk3, mlkl

## Abstract

Necroptosis, a recently discovered form of cell-programmed death that is distinct from apoptosis, has been confirmed to play a significant role in the pathogenesis of bacterial infections in various animal models. Necroptosis is advantageous to the host, but in some cases, it can be detrimental. To understand the impact of necroptosis on the pathogenesis of bacterial infections, we described the roles and molecular mechanisms of necroptosis caused by different bacterial infections in this review.

## 1 Introduction

Initially, researchers thought there were only two ways of cell death: apoptosis and necrosis. Apoptosis is active and program-controlled, and its key regulator is caspase. The morphological changes associated with apoptosis mainly include cell shrinkage and chromatin condensation, the formation of apoptotic bodies and cytoskeleton disintegration. Necrosis is passive and unprogrammed and can be activated in various ways, e.g., by bacterial infection (1), toxins (2), and ischemia (3); the morphological changes characteristic of necrosis include cellular swelling, plasma membrane rupture, and the release of cellular content (4, 5). In 2005, Professor Junying Yuan first demonstrated that nonapoptotic cell death induced by death receptor signaling is programmed (6), and coined the term “programmed necrosis” (also known as necroptosis). Necroptosis was originally named receptor-interacting protein 1 (RIP1)-dependent necrosis, referring to a caspase-independent form of programmed cell death that was characterized by both necrosis and apoptosis. During necroptosis, cells undergo changes such as membrane rupture, organelle swelling, and nuclear and cytoplasmic disintegration (7, 8).

**Figure 1:**
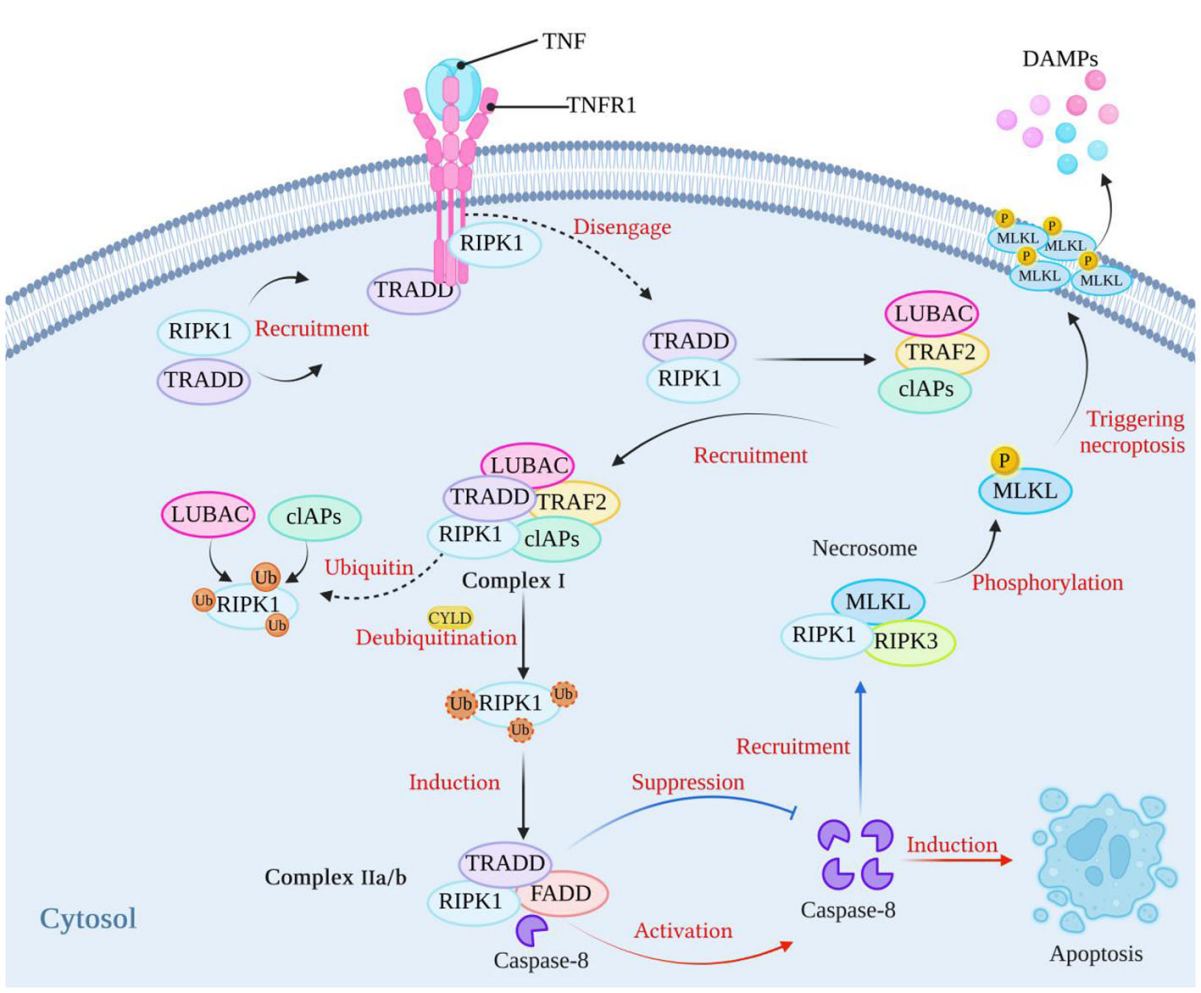
The classical mechanism of necroptosis. After TNF binds to its receptor, it can recruit RIPK1 and TRADD to form a complex. Subsequently, RIPK1 and TRADD dissociate from the TNF receptor and recruit LUBAC, TRAF2, and cIAPs to form complex I. At this stage, RIPK1 undergoes ubiquitination by LUBAC and cIAPs, which stabilizes the complex. When cIAPs are inhibited or when the ubiquitin chains on RIPK1 are removed using CYLD, a complex called complex IIa/b, consisting of TRADD, RIPK1, and FADD, is formed. Complex IIa/b activates caspase-8, which then induces cell apoptosis. When caspase-8 activity is blocked, cell apoptosis is inhibited. Subsequently, RIPK1 recruits RIPK3 through interactions between their RHIM domains. RIPK3 then recruits MLKL, forming necrosomes. MLKL is phosphorylated, and phosphorylated MLKL molecules aggregate and translocate to the plasma membrane, thereby triggering necroptosis. (Created with BioRender.com).

Necroptosis depends on the activity of receptor interacting protein kinase-1 (RIPK1), receptor interacting protein kinase-3 (RIPK3), and mixed lineage kinase domain-like protein (MLKL) (9–11). Necroptosis can be initiated by tumor necrosis factor receptor (TNFR) (12), pattern recognition receptors (PRRs), including Toll-like receptor (TLR3/4), Nod-like receptors (NLRs), and RIG-I-like receptors (RLRs) (4, 13–16), INF-α receptors (17, 18), adhesion receptors (19), and DNA-dependent activator of IFN (DAI) (also known as ZBP-1) (20, 21). The most classical pathway is TNF-induced necroptosis, which is described in **Figure 1**. After TNF binds to TNFR1 on the cell membrane, it recruits TNFR1-associated death domain protein (TRADD) and RIPK1 to form an early complex, which is subsequently detached from TNFR and recruits TNF receptor-associated factor 2 (TRAF2), cellular inhibitors of apoptosis (cIAPs) (including cIAP1/2) and linear ubiquitin chain assembly complex (LUBAC) to form complex I. At this time, RIPK1 is ubiquitinated by cIAPs and LUBAC, and the result of ubiquitination is the stabilization of complex I, which then continues to recruit downstream proteins, such as TGF activated kinase 1 (TAK1), TAK1 binding protein 2/3 (TAB2/3), and an IκB kinase complex (IKK) composed of IKKα, IKKβ, and NF-κB essential modulator (NEMO) (4, 22). The recruited downstream proteins activate the NF-κB and mitogen-activated protein kinase (MAPK) pathways, resulting in increased expression of proinflammatory genes, which contribute to the production of proinflammatory factors (4, 22). Blocking cIAPs or cylindromatosis protein (CYLD) to remove the ubiquitin chain on RIPK1 (23) can inhibit the ubiquitination of RIPK1 and induce the formation of complex IIa/b, which is composed of TRADD, RIPK1, and FAS-associated death domain protein (FADD). Complex IIa/b activates caspase-8 and then induces apoptosis (5, 24). RIPK1 contains an N-terminal kinase domain, a C-terminal death domain (DD), and an RIP homotypic interaction motif (RHIM). When the activity of caspase-8 is blocked and apoptosis is inhibited, RIPK1 recruits RIPK3 through interactions between the RHIM domains, and RIPK3 continues to recruit downstream MLKL and forms a complex, called a necrosome. RIPK3 recruits MLKL and phosphorylates MLKL, which subsequently translocates to the plasma membrane, triggers necroptosis, and releases damage-associated molecular patterns (DAMPs) from the cell (25–27), leading to excessive inflammation (28). However, the immune system has evolved RIPK1-independent necroptosis, and RHIM domain-containing protein molecules such as ZBP1 and TIR domain-containing adapter-inducing interferon-β (TRIF) can both induce necroptosis through the binding of their own RHIM domain to RIPK3 (20).

**Figure 2:**
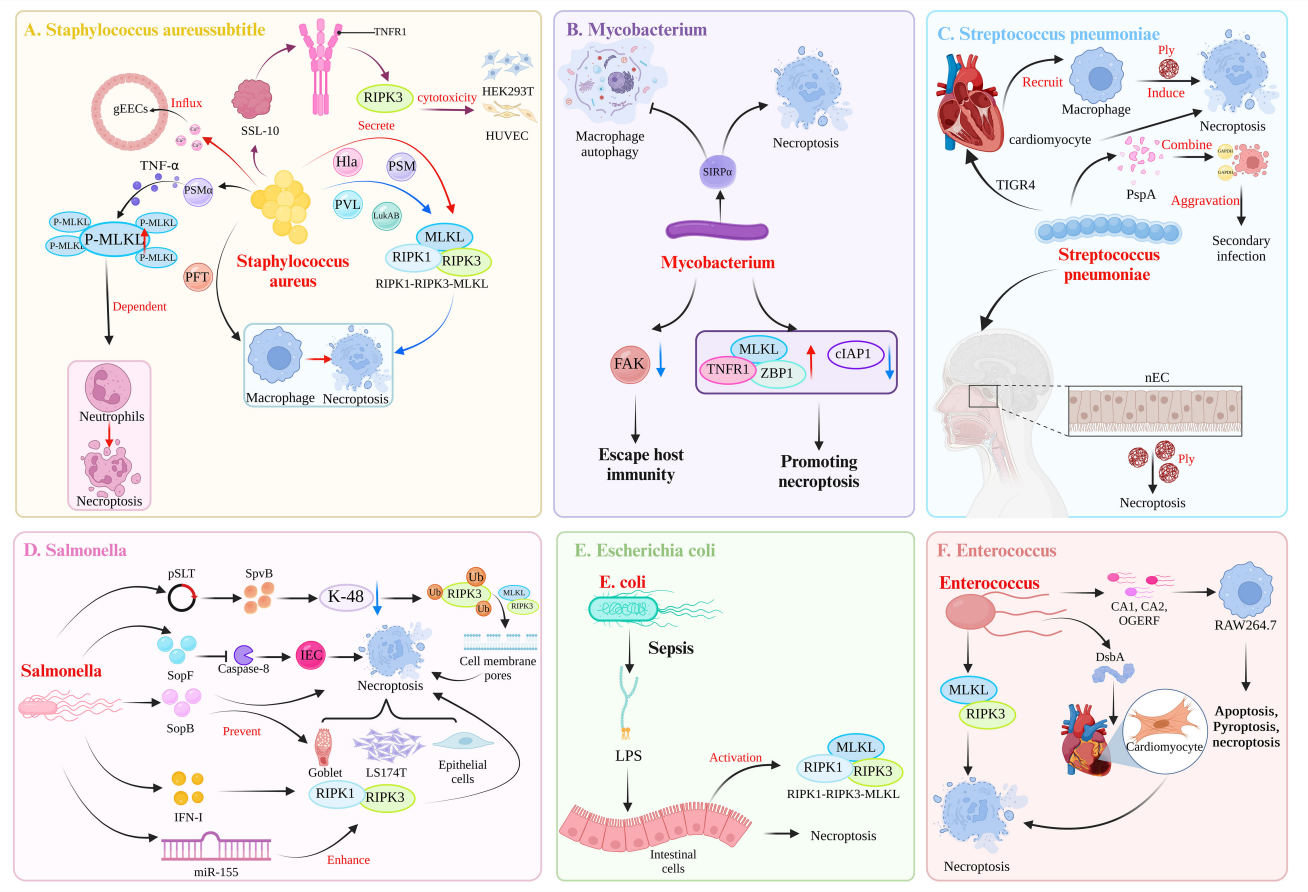
Mechanisms of bacterial induction of necroptosis. **(A)** Toxic virulence factors secreted by *Staphylococcus aureus*, such as Hla, PAM, PVL, and LukAB, can induce necroptosis in macrophages through the RIPK1-RIPK3-MLKL pathway. Among them, PSMα can induce TNFα secretion, leading to MLKL-dependent necroptosis in neutrophils. In addition, the virulence factor PFT can induce necroptosis in macrophages. The *S. aureus*-secreted SSL-10 binds to TNFR on the cell membrane to induce RIPK3-dependent necroptosis in HEK293T cell and HUVECs. *S. aureus* can induce necroptosis in gEECs, and the trigger is Ca^2+^ influx. **(B)** *Mycobacterium tuberculosis* triggers SIRPα in macrophages, leading to the inhibition of autophagy and the promotion of necroptosis. Additionally, *M. tuberculosis* can downregulate the expression of FAK in macrophages to evade host immunity. *M. tuberculosis* also shapes an environment that promotes necroptosis in macrophages by upregulating MLKL, TNFR1, ZBP1 expression and downregulating cIAP1 expression. **(C)** Following invasion of the heart by the TIGR4 strain of *Streptococcus pneumoniae*, the secretion of Ply can induce necroptosis in both cardiac myocytes and recruited macrophages. During the asymptomatic colonization of *S. pneumoniae* in the nasopharynx, Ply can induce necroptosis in nasopharyngeal epithelial cells (nECs). In the case of coinfection with influenza A virus and *S. pneumoniae*, the surface protein A (PspA) of *S. pneumoniae* acts as a cell adhesin and binds to GAPDH in dying cells, thereby increasing the localization of *S. pneumoniae* in the lower airways and exacerbating secondary infection following influenza. **(D)** The pSLT-encoded SpvB effector factor inhibits K-48-mediated ubiquitination of RIPK3, thereby mediating the formation of cell membrane pores through the RIPK3-MLKL pathway, resulting in necroptosis of intestinal epithelial cells (IECs). The effector factor SopF of the T3SS can induce necroptosis of IECs by blocking the activity of caspase-8, thereby enabling *Salmonella enterica* to spread to the intestinal lamina propria. SopB, encoded by SPI-1, can prevent the necroptosis of goblet cells, LS174T cells, and epithelial cells. *S. typhimurium* utilizes the host’s IFN-I response to induce necroptosis in macrophages mediated by RIPK1-RIPK3. Additionally, *S. typhimurium* induces the expression of miR-155, which promotes macrophages necroptosis. This effect is a result of miR-155 targeting of the RIPK1-RIPK3 pathway, which further promote apoptosis. **(E)** During sepsis, the lipopolysaccharide (LPS) by *Escherichia coli* can upregulate the expression of the necrosis-related proteins RIPK1, RIPK3, and MLKL, leading to intestinal epithelial cells necroptosis. **(F)** After infection with *Enterococcus faecalis*, DsbA can induce microinjury in the heart of *Caenorhabditis elegans*. Subsequently, at the site of microinjury in the heart, *E. faecalis* can induce the apoptosis and necroptosis of cardiomyocytes. In refractory apical periodontitis, *E. faecalis* can induce necroptosis in macrophages mediated by RIPK3-MLKL. Strains of *E. faecalis* isolated from root canals (CA1, CA2) and the OGERF strain can induce apoptosis, pyroptosis, and necroptosis in RAW264.7 macrophages. (Created with BioRender.com).

Dysregulated necroptosis can be involved in the occurrence of neurological diseases, such as Parkinson’s disease (29) and Alzheimer’s disease (30). In addition, necroptosis can play a different role in tumor diseases. For example, in gastric adenocarcinoma (31) and non-small cell lung cancer (32), the body can provide a favorable environment for the growth of cancer cells by downregulating the expression of proteins related to the necroptosis pathway. Therefore, activating necroptosis may be an effective anticancer strategy. However, some studies have also demonstrated that the upregulation of RIPK3 expression can also promote the occurrence of some tumor diseases, such as aggressive and recurrent breast cancer, which can promote the strong proliferation of cancer cells (33). This dual role of necroptosis in the body is also reflected in pathogen infection. Necroptosis is a growing concern in the pathogenesis of bacterial infections (34). Cell death is a common result of interactions between bacteria and hosts (35). Necroptosis is the lytic death of cells, is generally considered highly proinflammatory, and plays an important role in the pathogenesis of bacterial infections (4, 36, 37). Necroptosis may protect the host. Conversely, in some cases, necroptosis has an adverse effect on the host. Therefore, understanding the role of necroptosis in the process of bacterial infection and its mechanism of action in the defense against bacterial infection is important. This paper focuses on whether necroptosis during bacterial infection is beneficial or harmful and discusses the mechanism of action (**Figure 2**, **Table 1**).

**Table 1:** Mechanism of necroptosis in bacteria.

Bacteria	Virulence factor	Cell	Mechanism of necroptosis	references
*Staphylococcus aureus*	Hla,PSM,LukAB,PVL	Macrophage	Virulence factors induce necroptosis by activating RIPK1-RIPK3-MLKL pathway, thereby escaping host immunity, in which PFT induces necroptosis of macrophages through membrane rupture.	(36)
	PSMα	Neutrophil	PSMα induces the secretion of TNFα through FPR2 to trigger necroptosis.	(38)
	SSL-10	HEK293T,HUVEC	SSL-10 binds to TNFR1 on the cell membrane and triggers necroptosis through the RIPK1-RIPK3-MLKL and RIPK3-CaMKII-mPTP pathways.	(39)
	Unknown	gEECs	Necroptosis is induced by RIPK1-RIPK3-MLKL pathway, and its upstream triggering event is Ca^2+^ inflow.	(40)
*Mycobacterium tuberculosis*	Unknown	Macrophage	*M. tuberculosis* induces necroptosis of macrophages by activating SIRPα.	(41)
*Streptococcus pneumoniae*	Ply	Macrophage	The formation complex of RIPK3, RIPK1, MLKL and MCU induces mitochondrial calcium uptake and mROS production, and RIPK3 can initiate necroptosis through mROS mediating the opening of mPTP.	(42)
	Ply	nEC	During asymptomatic colonization of the nasopharynx by *S. pneumoniae*, nEC can die from Ply dependent necroptosis.	(43)
	PspA	Pulmonary epithelial cells	PspA binds to dead cells and increases the localization of *S. pneumoniae* in the lower airway.	(44)
*Streptococcus pneumoniae* (TIGR4 strain)	Ply	Macrophage	After TIGR4 strain invaded the heart, Ply caused necrptosis of macrophages.	(45)
*Salmonella*	Unknown	Macrophage	*Salmonella* uses host IFN-I response to induce RIPK1-RIPK3-mediated necroptosis.	(46, 47)
	SpvB	IEC	SpvB induces cell death via the RIPK3-MLKL pathway by down-regulating K-48-mediated RIPK3 ubiquitination.	(48)
	SopF	IEC	SopF blocks the activity of caspase-8 by activating the PDK1-RSK pathway, thereby promoting necroptosis, leading to bacterial spread and exacerbating systemic infection.	(49)
	SopB	goblet cell,IEC,LS174T	SopB protects cells from necroptosis, but causes bacteria to replicate in the cell and infect neighboring cells.	(50)
*Escherichia coli*	LPS	Intestinal cell	LPS induces cell necroptosis by up-regulating RIPK1-RIPK3-MLKL pathway-related proteins.	(51)
*Enterococcus faecalis*	DsbA	Myocardial cell	DsbA induced cardiac microlesions and MLKL-dependent necroptosis of cardiomyocytes.	(52)
	Unknown	Macrophage	In RAP lesions, *E. faecalis* induces RIPK3-MLKL mediated necroptosis of macrophages.	(53)
*Pseudomonas aeruginosa*	Unknown	Lung epithelial cells	*P. aeruginosa* mediates the necroptosis of lung epithelial cells,which can promote the change of mitochondrial membrane potential and thus release ROS.	(54)
*Listeria monocytogenes*	Unknown	IECs	*RIPK3^-/-^* mice had significantly increased *Listeria* proliferation and resulted in systemic infection.	(55)
*Francisella tularensis*	Unknown	Macrophage	Caspase-dependent apoptosis and RIPK1-RIPK3-dependent necroptosis can occur simultaneously in macrophages infected by *F. tulafera*.	(56)
*Shigella flexneri*	OspC1	IECs	The effector OspC1 blocks caspase-8 signaling to prevent apoptosis and subsequently triggers necroptosis as a host defense mechanism.	(57)

## 2 The role of necroptosis in bacterial infection

### 2.1 Staphylococcus aureus

*Staphylococcus aureus* is the main pathogen of nosocomial infections and can cause a variety of diseases, such as pneumonia, endocarditis, sepsis, and osteomyelitis, which seriously threaten human health (58). In the process of infection, in addition to being able to induce inflammatory cells to release inflammatory factors and accumulate at the site of infection to trigger an inflammatory response (59–63), *S. aureus* can also induce necroptosis in host cells, such as macrophages (36) and neutrophils (38).

A typical feature of *S. aureus* pneumonia is toxin-induced necroptosis of immune and resident cells. Virulence factors secreted by *S. aureus*, such as Hla, PSM, LukAB and PVL, can induce necroptosis in macrophages through the RIPK1-RIPK3-MLKL pathway (36). PSMα reportedly triggers neutrophil necroptosis via FPR2-induced TNFα secretion (38). Some *S. aureus* phagocytosed by polymorphonuclear neutrophils (PMN) can survive in phagosomes, thereby inducing PMN necroptosis, and this process is dependent on RIPK3 and independent of RIPK1 and MLKL (64, 65). Examples of necroptosis independent of active RIPK1 or MLKL have been reported. TLR3 or TLR4 can activate RIPK3 through TRIF, thereby directly initiating necroptosis through TRIF-RIPK3-MLKL (16). RIPK3 can also induce necroptosis of cardiomyocytes through calmodulin-dependent protein kinase II (CaMKII) and mitochondrial pathways (66). Thus, delineating the molecular pathways that ultimately lead to PMN death will provide new targets for the treatment of *S. aureus* infections. After *S. aureus* is phagocytosed by macrophages, approximately 10% of the *S. aureus* population will persist in macrophages, with the remaining bacteria eliminated by bacteriolysis. However, excessive bacteriolysis can cause cell death (67). Further studies confirmed that this type of cell death is not apoptosis or pyroptosis (It is characterized by dependence on inflammatory caspase enzymes, mainly caspase-1, 4, 5, 11, accompanied by the release of a large number of pro-inflammatory cytokines) but rather AIM2-mediated necroptosis (67). Apoptosis is known to be important for clearing pathogens (68), and the authors propose a potential immune manipulation strategy by which S. aureus sacrifices the minority to trigger a limited necroptosis, thereby releasing signals from dead cells to inhibit apoptosis and other anti-inflammatory cascades of live cells, eventually surviving within host cells and establishing infection (67). In addition to AIM2, there are other pattern recognition receptors that mediate necroptosis, such as NLRC4, which inhibits the IL-17A-dependent recruitment of neutrophils by upregulating IL-18 expression and inducing necroptosis during *S. aureus* pneumonia. Loss of NLRC4 signaling contributes to host protection against *S. aureus* pneumonia, and treatment with necroptosis inhibitors or IL-18 gene ablation has been shown to enhance defenses against *S. aureus* in mice (69). Therefore, modulating the function of NLRC4 may be a potential approach for the treatment of *S. aureus* infection. However, stimulator of interferon genes (STING), which is an intracellular pattern recognition receptor (70), enhances the host response to *S. aureus* infection by inhibiting the necroptosis of macrophages.

In addition to immune cells, *S. aureus*-induced necroptosis is also observed in some nonimmune cells. When the human lung epithelial cell line A549 was infected with *S. aureus*, TNFα enhanced pulmonary epithelial cell injury by *S. aureus*, and the mechanism is related to RIPK3-mediated necroptosis (71). *S. aureus* secretes the toxic protein *staphylococcal* superantigen-like protein-10 (SSL-10), which has been shown to interfere with host cell inflammatory responses by binding to ERK2 (72), but whether SSL family proteins can induce cytotoxicity remains unknown. However, a recent study showed that SSL-10 can bind to the receptor TNFR1 on the cell membrane and exert strong cytotoxic effects in two types of nonimmune cells, HEK293T cells and HUVECs, by inducing RIPK3-dependent necroptosis; this study also showed that necroptosis is activated by two distinct signaling pathways, the RIPK1-RIPK3-MLKL and RIPK3-CaMKII mitochondrial permeability transport pore (mPTP) pathways (39). This study described the cytotoxicity of the SSL-10 protein, an inducer of necroptosis, and provided a potential target for the clinical treatment of *S. aureus*-related diseases (39). However, whether other proteins in the SSL family have the same effect remains to be further investigated. Furthermore, in another nonimmune cell type, *S. aureus*-induced necroptosis played a pathological role, and *S. aureus*-induced goat endometrial epithelial cells (gEECs), by increasing the expression of key proteins in the RIPK1-RIPK3-MLKL pathway, induced significant necroptosis. The authors found that the inducers of necroptosis in gEECs were not traditional TLRs or TNFRs but membrane disruption and ion imbalances (40), that is, Ca^2+^ influx; furthermore, blocking membrane permeability with glycine protected gEECs from *S. aureus*-induced cell death. Necroptosis initiated during *S. aureus* infection is mostly detrimental to the host; therefore, inhibitors targeting necroptosis may be an effective strategy for the treatment of *S. aureus* infection. In a recent report, a salt-inducible kinase (SIK) inhibitor, HG-9–91-01, was found to block necroptosis by inhibiting RIPK3 activity, thereby attenuating necroptosis-mediated inflammatory damage (73). However, the consequences of necroptosis are not necessarily proinflammatory. Necroptosis results in the elimination of cells that produce cytokines and inflammatory products, and therefore the bacteria inside the cells are released. These bacteria to be eliminated by neutrophils, leading to an overall reduction in inflammation (74, 75). In a previous study, necroptosis was found to play an immunoprotective role in a skin model of *S. aureus* infection, not by participating in cell death but by limiting damage caused by excessive inflammation (76). In the authors’ study, failure to activate necroptosis was associated with excessive local pathology and impaired *S. aureus* clearance. Thus, necroptosis contributes to host recovery after *S. aureus* infection.

### 2.2 Mycobacterium tuberculosis

Over a quarter of the world’s population is infected with *Mycobacterium tuberculosis* (Mtb), the causative agent of tuberculosis (TB). TB remains a major burden on global public health (77). Understanding the host response to *M. tuberculosis* infection is a key aspect of eradicating TB through the development of effective vaccines and immunotherapies. During infection, *M. tuberculosis* can trigger substances in macrophages to damage the body. Signal regulatory protein alpha (SIRPα), which is mainly expressed in myeloid cells, such as monocytes/macrophages and dendritic cells (78), can participate in the pathogenesis of *M. tuberculosis*. The cytoplasmic region of SIRPα contains four immunoreceptor tyrosine-based inhibitory motifs (ITIMs) that, upon ligand binding, become phosphorylated and interact with the SH2-domain-containing tyrosine phosphatases (PTPase) SHP-1 and SHP-2 to mediate various biological functions (79). In the process of *M. tuberculosis* infection, SIRPα can inhibit the autophagy of macrophages and promote the necroptosis of macrophages. The occurrence of necroptosis is the main way that affects the weakening of the killing ability of macrophages against *M. tuberculosis*. The tail structure of SIRPα in the cell can directly bind to protein tyrosine kinase 2B (PTK2B) to affect the activity of PTK2B. When SIRPα is knocked out, it can promote the binding of SHP-1 to PTK2B, resulting in the activation of PTK2B. Therefore, PTK2B can bind to the death domain of RIPK1 to inhibit necroptosis of macrophages (41). The bactericidal capacity of macrophages was enhanced when necroptosis was blocked with the RIPK1 inhibitor NEC-1 (41). The results of this study suggest that targeting SIRPα may become a new therapy for the treatment of *M. tuberculosis*. However, studies have shown that SIRPα mutant mice have increased susceptibility to *Salmonella typhimurium* infection, suggesting that SIRPα may help the host defend against infection by the pathogen (80). Therefore, the role of SIRPα in host defense against pathogen infection is complex and requires further exploration. In addition, focal adhesion kinase (FAK) in human macrophages can block the occurrence of *M. tuberculosis*-induced macrophage necroptosis and *M. tuberculosis* can downregulate FAK expression, thereby evading host immunity (81). *M. tuberculosis* can also shape the environment to promote macrophage necroptosis by upregulating MLKL, TNFR1, and ZBP1 expression and downregulating cIAP1 expression (82). However, blocking necroptosis by knocking out the *mlkl* gene or inhibiting RIPK1 had no effect on the survival of infected human or mouse macrophages (82). Taken together, these results indicate that the inhibition of macrophage necroptosis may alleviate disease pathogenesis after *M. tuberculosis* infection, but not change the outcome of the disease.

### 2.3 Streptococcus pneumoniae

*Streptococcus pneumoniae* is a gram-positive bacterium that often colonizes the nasopharynx (83), and asymptomatic bacterial colonization can cause invasive diseases, such as pneumonia and meningitis (84), under certain conditions. *S. pneumoniae* carries two key virulence proteins, including pneumococcal surface protein A (PspA) and pulmonary hemolysin (Ply) (85, 86). RIPK3 is considered a key regulator of inflammation and cell death, and significantly elevated RIPK3 protein concentrations have been detected in patients with *S. pneumoniae* pneumonia (42). Further studies showed that RIPK3, RIPK1, MLKL and the mitochondrial calcium uniporter (MCU) combine to form complexes during *S. pneumoniae* infection, inducing mitochondrial calcium uptake and mROS production (42). In macrophages, RIPK3 can mediate mitochondrial permeability transport pore opening through mROS to initiate necroptosis and activate the NLRP3 inflammasome (inflammasome is a multiprotein complex assembled by cytoplasmic PRRs, inflammasome can recognize PAMPs or DAMPs, recruit and activate the proinflammatory protease caspase-1.) through the mROS-AKT pathway to protect against *S. pneumoniae* invasion (42). Furthermore, during asymptomatic colonization of the nasopharynx by *S. pneumoniae*, nasopharyngeal epithelial cells (nECs) die via Ply dependent necroptosis. When the *mlkl* gene was knocked out and bacteria were colonized, or wild-type mice were colonized with *ply* deficient strains, mice had reduced production of the antibody against the bacterial surface protein PspA, delayed bacterial clearance, and increased vulnerability to secondary attack by *S. pneumoniae* (43). Therefore, Ply induced necroptosis contributes to the protective immunity of the host. *S. pneumoniae* can invade the heart after causing bacteremia, both the TIGR4 and D39 strains can effectively invade the myocardium, and macrophages recruited after TIGR4 invades the heart die from pneumolysin-induced necroptosis (45). However, whether neutralizing pulmonary hemolysin or blocking necroptosis is beneficial to the host remains unclear. In a recent study, the TIGR4 strain induced necroptosis in cardiomyocytes after invading the heart, a process mediated by Ply, and treatment with a necroptosis inhibitor reduced pneumonia in heart streptococcal lesions and decreased serum troponin levels (87). Therefore, the cardiac injury that occurs during invasive pneumococcal disease is due in part to cardiomyocyte necroptosis, and necroptosis inhibitors may be an effective treatment. Coinfection with influenza A virus (IAV) and *S. pneumoniae* leads to high mortality (88). IAV infection can promote the translocation of *S. pneumoniae* to the heart, and IAV can promote the adhesion of *S. pneumoniae* to cardiomyocytes by upregulating the expression of cardiomyocyte adhesion factors. In an *in vitro* model of cardiomyocyte infection, IAV enhanced Ply induced necrotic cell death by promoting oxidative stress, thereby increasing *S. pneumoniae* cytotoxicity (89). IAV infection can cause the death of lung epithelial cells through apoptosis, pyroptosis and necroptosis. Recently, PspA was reported to act as a cytoadhesin and bind to GAPDH in dead cells, thereby increasing the localization of *S. pneumoniae* in the lower airways and exacerbating secondary infection after influenza infection (44). This finding also helps explain why IAV patients have an increased susceptibility to *pneumococcal* infection.

### 2.4 Salmonella

*Salmonella enterica* is one of the leading causes of bacterial gastrointestinal infections in humans and animals (90); *Salmonella* can be transmitted to humans and animals via the fecal-oral route, and the gastrointestinal tract is the first site of host–pathogen interaction after the ingestion of *Salmonella*. *Salmonella* virulence factors mainly include pathogenicity islands, virulence plasmids, enterotoxins and endotoxins (91, 92). Pathogenicity islands are directly related to bacterial invasiveness, especially for SPI-1 and SPI-2. SPI-1 is essential for nonphagocytic invasion and is responsible for Salmonella-induced inflammation in colitis, whereas SPI-2 is essential for bacterial survival and proliferation in phagocytes and plays an important role in systemic infection (93, 94). The type III secretion system (T3SS) encoded by SPI-2 is the most widely studied and important virulence factor in *Salmonella*. After *Salmonella* enters the host, it uses the T3SS to secrete effectors into host cells. These effectors can promote bacterial colonization and intracellular survival (95). Therefore, *Salmonella* can successfully localize to the gut and infect a variety of cell types, such as intestinal epithelial cells and macrophages (95).

A key virulence strategy of *Salmonella typhimurium* is the induction of macrophage death. *S. typhimurium* can exploit the host’s type I IFN (IFN-I) response to eliminate macrophages by inducing RIPK1-RIPK3-mediated necroptosis (46). In this process, IFN-I activates RIPK3, which in turn impairs the Nrf2-dependent antioxidant stress response, thereby enhancing necroptosis in macrophages (47). In addition, *S. typhimurium*-induced miR-155 also enhances necroptosis in macrophages by targeting RIPK1-RIPK3 (96). Intestinal epithelial cells (IECs) and the microbiota in the intestinal mucosa form a barrier to protect against invasion by foreign pathogens (97), and when intestinal epithelial cells are damaged, pathological damage to the intestine can be induced. Recently reports show that *Salmonella* can disrupt the integrity of the intestinal epithelial barrier by inducing IECs necroptosis, thereby promoting the invasion of *Salmonella* into the intestine. In this process, the virulence plasmid of *S. typhimurium* (pSLT) that encodes the SpvB effector plays a crucial role. SpvB mediates the formation of cell membrane pores via the RIPK3-MLKL pathway by inhibiting K-48-mediated RIPK3 ubiquitination to achieve cell death (48). This process is considered RIPK1-independent necroptosis because cell death still occurred after the use of an RIPK1 inhibitor (48). SopF is a newly discovered T3SS effector that promotes bacterial dissemination in mice (98). During *S. typhimurium* infection, SopF blocks the activity of caspase-8, which is defined as the molecular switch for PANoptosis. When the activity of caspase-8 is blocked, it can inhibit apoptosis and pyrodeath of IEC cells, and promote IEC necroptosis of IEC (49). Therefore, necroptosis may related to the *S.typhimurium* to spread the lamina propria and cause systemic infection. Further studies revealed that SopF, as a phosphoinositide (PIP)-binding effector, can block caspase-8 by activating the 3-phosphoinositide-dependent protein kinase 1 (PDK1)-ribosomal S6 kinase (RSK) signaling pathway and, after treatment with AR-12 (PDK1 inhibitor) and BI-D1870 (RSK inhibitor), can reverse abnormal apoptosis, pyroptosis and necroptosis (49). In addition, *Salmonella* exoprotein B (SopB), encoded by SPI-1, plays an important role in the *Salmonella* infection. Studies have shown that SopB is responsible for cell invasion after *Salmonella* infection (99). In contrast to SpvB-induced necroptosis, SopB plays a role in preventing necroptosis; for example, SopB can prevent necroptosis in nonimmune cells such as goblet cells, LS174T cells, and epithelial cells. Necroptosis in cecal goblet cells, LS174T cells, and epithelial cells can be promoted by increasing MLKL phosphorylation after infection with SopB-deficient strains (50). However, even though the presence of SopB protects epithelial cells from necroptosis, it also allows *Salmonella* to replicate in epithelial cells, which subsequently promotes bacterial escape from epithelial cells and increases their ability to infect neighboring cells, which may play a role in bacterial dissemination (50). In addition, SseK1 and SseK3 inhibited NF-κB activation and necrotizing apoptosis during *Salmonella* infection of macrophages in a study of the T3SS effector protein SseK (100). Inhibition of both proinflammatory signaling and host cell death by SseK1 and SseK3 may be a strategy that *Salmonella* plays to reproduce in host cells, thus providing *Salmonella* with robustness and flexibility in counteracting host immune responses.

However, many studies have also shown that *Salmonella* does not activate necroptosis. Non-invasive *S. typhimurium* does not naturally induce RIPK3-dependent macrophage death, whereas macrophage necroptosis can only be induced when caspase is inhibited using Z-VAD-FMK, and RIPK3 induction (after caspase inhibition) does not affect host survival after systemic *Salmonella* infection (101). Furthermore, the induction of RIPK3 leads to the recruitment of hypoinflammatory myeloid cells, contrary to the usual characterization of necroptosis as highly proinflammatory. Similarly, the synergistic role of RIPK3 and caspase-3/11 in regulating *Salmonella* burden *in vivo* was described in another study (102). Furthermore, mice with previously known caspase-3/11 deletions were shown to have an impaired ability to control the *Salmonella* burden, whereas RIPK3 deletion alone did not affect the innate immune response to *Salmonella* infection (102). In addition, studies in recent years have shown that single deletion of cell death, apoptosis, or necroptosis has little effect on *Salmonella* control, and that combination of these cell death pathways leads to loss of bacterial control in mice and their macrophages (103).

### 2.5 Escherichia coli

*Escherichia coli* is a common gram-negative bacterium with many virulence factors, including endotoxin, a capsule, a type III secretion system, adhesin and exotoxins; exotoxins include Shiga toxin, thermostable enterotoxin and heat labile enterotoxin (104). When these virulence factors are present in different types of *E. coli*, they have different pathogeneses and disease outcomes. Lipopolysaccharide (LPS) is a component of the outer cell wall of gram-negative bacteria, and LPS is an endotoxin that has toxic effects on the host. LPS induces necroptosis in host cells to promote disease (105). During sepsis, *E. coli* LPS can induce the intestinal cell necroptosis, activate the necroptosis signaling pathway, and upregulate the expression of the necrosis-related proteins RIPK1, RIPK3, and MLKL; furthermore, LPS is associated with intestinal morphology and functional damage (51). Pretreatment with the RIPK1 inhibitor NEC-1 reduces the extent of ultrastructural changes caused by necroptosis (51). Therefore, NEC-1 may prevent some intestinal damage during sepsis. Intestinal epithelial cell injury and inflammation can also be induced by enterotoxigenic *E. coli* (ETEC), but unsaturated fatty acids (EPA) and arachidonic acid (ARA) can alleviate enterotoxigenic *E. coli* induced intestinal damage by modulating necroptosis signals (106) because EPA and ARA inhibit the expression of the RIPK1, RIPK3, and MLKL proteins. The T3SS effectors NleB and EspL of EPEC block cell necroptosis; NleB works by inactivating the death domains of proteins, including TRADD, FADD, RIPK1, and TNFR1, to block TNFα-induced necroptosis (107). EspL inhibits TNF-induced necroptosis by cleaving the RHIM domains of RIPK1, RIPK3, TRIF and ZBP1/DAI (108). Therefore, the inhibition of necroptosis contributes to the continued colonization of EPEC *in vivo*, thereby contributing to disease progression.

### 2.6 Enterococcus faecalis

*Enterococcus faecalis* is a common gram-positive bacterium and part of the normal flora in the intestinal tract of animals. Generally, *E. faecalis* is harmless to humans and animals. However, recent studies have shown that some *E. faecalis* strains have evolved more virulence genes that allow them to infect the human body and cause a variety of diseases, such as endocarditis and peritonitis (109). Disulfide bonding protein A (DsbA) was found to be essential for fecal enterococcal virulence in a model of *E. faecalis* infection of the Shirley Cryptococcus nematode. DsbA can cause microdamage during heart formation. Subsequently, at the site of cardiac microinjury, *E. faecalis* can induce cardiomyocyte apoptosis and necroptosis, which in turn contribute to cardiac microinjury (52); however, although the EntV protein is a substrate of DsbA, in this study, the absence of EntV did not alleviate the symptoms of the disease, and therefore, future studies should explore and identify other substrates of DsbA to determine whether they contribute to cardiac micropathology. In addition, in immune cell studies, root canal isolates (CA1 and CA2) and OGERF induced upregulated expression of RAW264.7 macrophage apoptosis-related proteins associated with pyroptosis and necroptosis (110). In refractory apical periodontitis, *E. faecalis* can induce RIPK3-MLKL-mediated necroptosis in macrophages. This study suggested that inhibitors or treatments targeting necroptosis are a viable strategy for the treatment of refractory apical periodontitis (53). Notably, the increase in MLKL phosphorylation does not necessarily indicate necroptosis (111).

### 2.7 Other bacteria

#### 2.7.1 Pseudomonas aeruginosa

Acute lung injury caused by *Pseudomonas aeruginosa* is a disease that seriously endangers public health. Recent reports indicated that *P. aeruginosa*-mediated necroptosis of epithelial cells plays an important role in this pathological process. *P. aeruginosa* mediated acute lung injury and lung inflammation can be alleviated by inhibiting the necroptosis pathway. Moreover, the NLRP3 inflammasome is involved in this pathological process, and MLKL-dependent necroptosis signaling can promote changes in mitochondrial membrane potential, thereby releasing reactive oxygen species (ROS), which are important triggers for inflammasome activation (54). In addition, in a recent study showing that the RIPK3 scaffold plays a regulatory role in lung inflammation during *P. aeruginosa* infection, blocking the RHIM domain in RIPK3 with M45 reduced the inflammatory response to infection *in vitro* (112). Therefore, the inhibition of RHIM signaling is a potential strategy for reducing lung inflammation during infection.

### 2.7.2 Listeria monocytogenes

As an important mediator of necroptosis, RIPK3 can be abundantly expressed in the gastrointestinal; and after oral infection with *Listeria monocytogenes*, compared with wild-type mice, *RIPK3^-/-^* mice exhibited significantly increased *Listeria* proliferation, resulting in systemic infection (55). Moreover, Studies have shown that the activation of MLKL induced by *Listeria* infection does not cause intestinal epithelial cell necroptosis, on the contrary, MLKL can directly bind to *Listeria*, thereby inhibiting pathogen replication (55). This finding illustrates the importance of necroptosis in the defense against bacterial infection. However, in *Listeria*-induced acute liver injury, the inhibition of necroptosis significantly ameliorated mitochondrial dysfunction in mouse livers (113). In addition, *Listeria* infection can also rapidly induce necroptosis of macrophages (114, 115).

### 2.7.3 Francisella tularensis

*Francisella tularensis* is the pathogen responsible for tularemia, and the infection of host cells induces host cell death (116). Caspase-dependent apoptosis and RIPK1-RIPK3-dependent necroptosis can occur simultaneously in macrophages infected by *F. tularensis*, and the presence of z-VAD-FMK (caspase inhibitor) and NEC-1 significantly reduces the level of cell death (56). What has not been fully explained, however, is how cell necroptosis is initiated in the early (< 72h) period of tularemia without TNF production (56). We believe that this understanding of TNF independent necroptosis mechanisms could help identify drug targets.

### 2.7.4 Shigella flexneri

*Shigella flexneri* is a pathogen responsible for bacillary dysentery that can invade and colonize the intestinal epithelial cells of the host, eventually leading to severe inflammatory colitis. Cell death is thought to be a key aspect of host resistance to bacterial invasion (117, 118). However, in *Shigella*-infected intestinal epithelial cells, no cell death was observed (119). A recent study demonstrated that effectors of *Shigella’s* T3SS play a crucial role in blocking host cell death. After *Shigella* infects intestinal epithelial cells, the effector OspC1 blocks caspase-8 signaling to prevent apoptosis and subsequently triggers necroptosis as a host defense mechanism (57). However, to counteract the host response to bacterial infection, *Shigella* employs OspD3 to target and cleave the RHIM domains of RIPK1 and RIPK3, thereby degrading RIPK1 and RIPK3 and inhibiting necroptosis in host cells (57). Therefore, this phenomenon of “cell death cross-talk” promotes the survival and proliferation of Shigella within host cells.

## 3 Conclusion and opinions

Currently, the emergence of multidrug-resistant bacteria, such as *methicillin-resistant Staphylococcus aureus* (120), has become a focus of concern; therefore, finding more effective targets to fight infection by pathogens is critical. In this article, we describe the roles and molecular mechanisms of necroptosis caused by different bacterial infections. In recent years, a wealth of evidence has indicated that necroptosis plays a significant role in various types of bacteria. Bacteria can utilize their own virulence factors or alter the composition of host cells to activate or inhibit necroptosis. These effects can benefit the host (42) or harm the host (105). Therefore, unraveling the role of necroptosis in different bacterial infections is crucial. Here, we review the current understanding of necroptosis in bacterial infection. More detailed information is provided for *S. aureus*, *M. tuberculosis*, *S. pneumoniae*, *Salmonella*, *E. coli*, and *E. faecalis* in this review because of the comprehensive or controversial functions of necroptosis reported in these bacteria compared with other bacteria.

After bacteria invade the host, necroptosis mainly occurs in two major types of cells. One is immune cells that play a phagocytic role after bacteria enter the body. In this article, we mainly focus on neutrophils and macrophages because they are the main cells that play a role in the early stages of bacterial infection. The other is resident cell at the site of bacterial invasion or colonization.

Neutrophils and macrophages are important components of the natural immune system. Neutrophils are the most abundant white blood cells in the systemic circulation, that can exert bactericidal effects through both oxygen-dependent pathways (myeloperoxidase, MPO) and oxygen-independent pathways (antimicrobial peptides and proteins) (121). Myeloperoxidase (MPO) is released from primary particles, and produces reactive oxygen species (ROS). Primary particles can also release antimicrobial peptides, such as defensins. Secondary particles can release antimicrobial proteins, such as lysozyme. Bacteria have evolved multiple strategies to interfere with the bactericidal mechanism of neutrophils. Inducing the death of neutrophils is one of these strategies, and in this article, we mainly focus on necroptosis as a programmed cell death pathway. For example, *S. aureus* induces necroptosis in neutrophils by releasing its virulence factor, PSMα (38). Macrophages have a range of mechanisms to clear pathogens, including the release of reactive oxygen species (ROS), active nitrogen (RNS), enzymes and antimicrobial peptides, as well as the acidification of phagolysosomes, nutrient restriction and autophagy (62). Similarly, bacteria can evade macrophage killing through a variety of strategies. *S. aureus* induces necroptosis in macrophages through its virulence factors Hla, PSM, LukAB and PVL (36). In addition, *Salmonella* (46) and *E. faecalis* (53) also induce necroptosis of macrophages to interfere with bacterial clearance by macrophages. Taken together, these examples suggest that immune cell death is detrimental to the host and that inhibiting key proteins associated with necroptosis to prevent immune cell death may be an effective strategy for treating bacterial infection. In addition, signaling regulatory protein alpha (SIRPα), which is expressed mainly on the surface of myeloid cells, may be an important target for the treatment of *M. tuberculosis* infection. SIRPα promotes the necroptosis of macrophages to reduce their ability to kill *M. tuberculosis* (41). However, mice with SIRPα deficiency showed increased susceptibility to *S. typhimurium* infection, indicating that SIRPα may contribute to host defense against Salmonella infection (80). We speculate that the different effects of SIRPα on the host may be related to the different gram-negative nature of the bacteria.

Research has shown that the occurrence of necroptosis depends on the activity of RIPK1, RIPK3, and MLKL. RIPK3, a key protein in the necroptotic pathway, induces necroptosis in macrophages during *S. pneumoniae* infection and activates the NLRP3 inflammasome in response to *S. pneumoniae* infection (42). RIPK3 regulates the balance between inflammatory signaling, which promotes bacterial clearance, and the lethal consequences of excessive inflammation. RIPK3 activates the NLRP3 inflammasome to secrete proinflammatory cytokines, thereby inducing immune cell recruitment and bacterial clearance while also regulating necroptosis by clearing dead bacteria and cell debris to prevent excessive inflammation and maintain immune homeostasis (42). In recent years, there have been many reports of macrophages necroptosis induced by bacterial infection. However, neutrophils, as the most common circulating white blood cells in the body, are rarely reported to undergo induce necroptosis after bacterial infection. This distinction may be due to macrophages expressing a wide range of pattern recognition receptors, which can trigger a variety of bacterial sensing systems that induce necroptosis.

Epithelial cells are cells on the surface of the skin or lumen that maintain tissue function by forming a barrier while also participating in the immune response. Epithelial cell death caused by bacterial invasion of the host may lead to barrier destruction, thus facilitating bacterial invasion. For example, *S. aureus* can induce necroptosis in gEECs (40). *Salmonella* and *E. coli* can cause IEC death after intestinal invasion (48, 51). The process of *Salmonella* infection in epithelial cells is complex. To establish infection, *Salmonella* enters and replicates in epithelial cells and subsequently escapes from them with the help of effectors (122). Here, the authors propose that *Salmonella* has a time-regulating ability. In the initial stages of infection, bacteria must prevent cell death; for example, SopB, encoded by *Salmonella* SPI-1, protects epithelial cells from necroptosis (50), thus providing a favorable environment for bacteria to replicate. In the final stage of infection, cell death is needed to promote *Salmonella* escape from epithelial cells, a role reversal that requires the time regulation of effector proteins (50). The ability of bacteria to regulate time may provide a theoretical basis for understanding the pathogenesis of bacteria.

Successful control of *S. aureus* infection requires two major host responses: rapid suppression of *S. aureus* replication and rapid regulation of the subsequent excessive inflammatory response (75, 123). Therefore, we speculate that the dual role of necroptosis after bacterial infection is related to the two factors. On the one hand, cells can provide a favorable environment for bacteria to replicate, and properly triggering necroptosis can inhibit bacterial replication. However, excessive cell death may cause a breakdown of the host barrier and allow for the release of bacteria, which are then internalized by macrophages and spread throughout the body. Alternatively, excessive immune cell death allows the bacteria to escape host immunity. On the other hand, necroptosis may be involved in regulating the balance between proinflammatory signals and excessive inflammation, which can lead to the death of cells that produce cytokines and inflammatory products, thus limiting excessive inflammation. For example, necroptosis played a protective role in a model of *S. aureus* skin infection by limiting excessive inflammation (76). This imbalance may be related to the type of bacteria and the severity of the infection. Overall, exploring the beneficial and detrimental effects of necroptosis on the host is helpful for identifying effective strategies for the treatment of bacterial infections. Furthermore, research into the mechanisms and physiological effects of necrotic apoptosis is needed to understand the effectors that target cell death, which may trigger cancer cell death or an anticancer immune response.

## Author contributions

XY: Writing – original draft. JY: Writing – original draft. LS: Writing – original draft. SD: Writing – original draft. LY: Writing – review & editing. MY: Writing – review & editing.
